# Supplementary material for: An 8000 years old genome reveals the Neolithic origin of the zoonosis Brucella melitensis
Source: Nat Commun. 2024 Jul 20;15:6132. doi: 10.1038/s41467-024-50536-1 (PMC11271283; doi:10.1038/s41467-024-50536-1)
Supplement: Supplementary file 1 — Supplementary Information [file 41467_2024_50536_MOESM1_ESM.pdf]

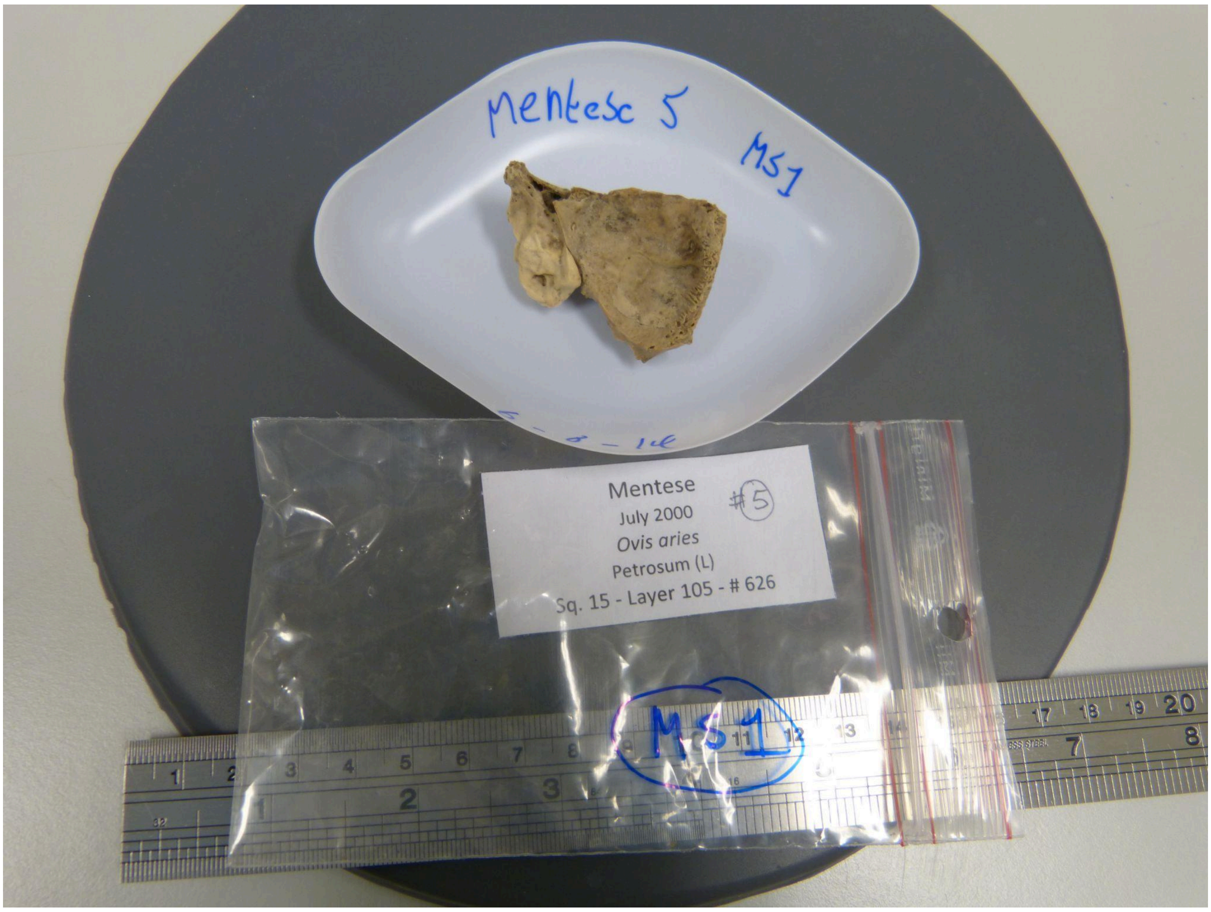

Supplementary Figure 1: Photo of the petrosal bone Mentese6. “MS1” refers to initial coding assigned with the archaeological identification. “Mentese5” refers to a deprecated labelling system.

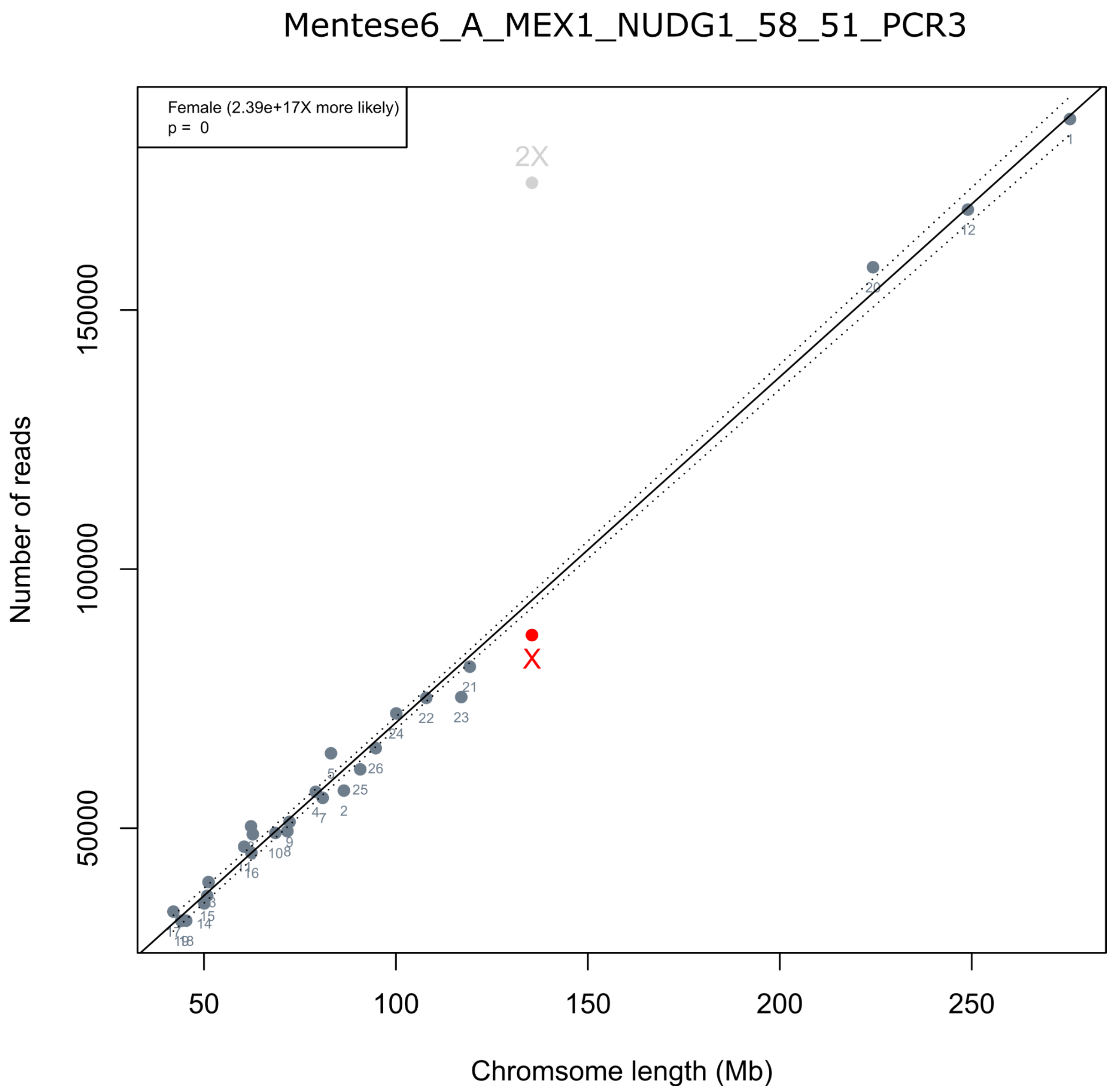

Supplementary Figure 2: Karyotypic sex based on the ratio of reads aligning to chromosomes and chromosome length. The Mentese6 specimen has roughly the number of reads aligning to the X chromosome expected, given the chromosome length. This suggests the sheep had two copies of the X chromosome and thus was karyotypic female

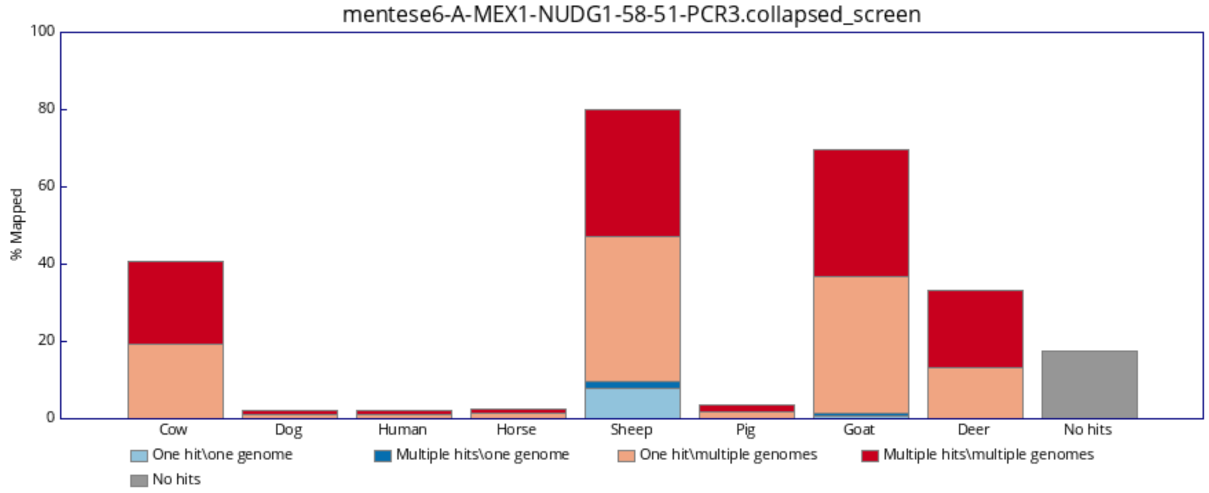

Supplementary Figure 3: Species identification of Mentese6. The bar plots represent the percentage of reads aligning with bowtie2 on different species reference genomes after a downsampling of 100,000 of reads from the total library. The red colour is representing the percentage of reads who are mapping on multiple positions along multiple genomes. Orange colour represents the percentage of reads who are mapping on only one position along multiple genomes. Dark blue represents the percentage of reads who are mapping on multiple positions on only one genome. Light blue represents the percentage of reads who are mapping one position on only one genome. Sheep has the highest total reads aligning and “One Hit One Genome” statistic, indicating the Mentese6 bone is from a sheep.

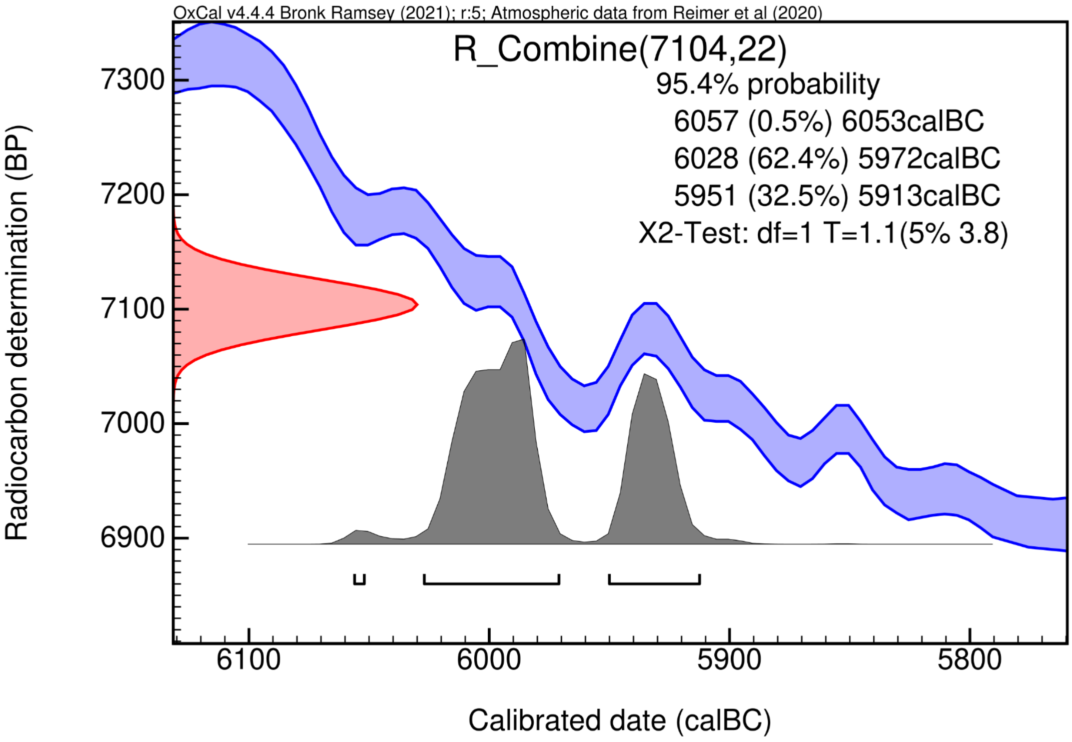

Supplementary Figure 4: Combined radiocarbon age estimate of Mentese6, calculated using Oxcal4.4. The two dates are internally consistent ($\chi 2 = 1.1$, 5% cutoff = 3.8).

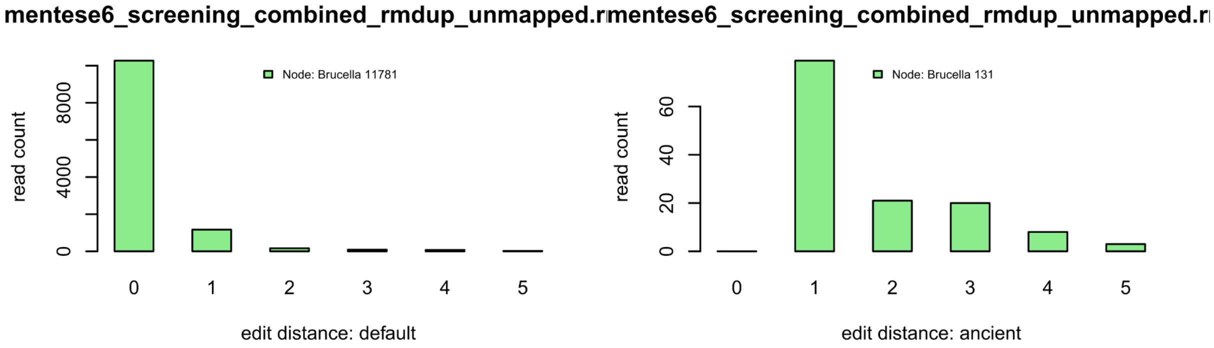

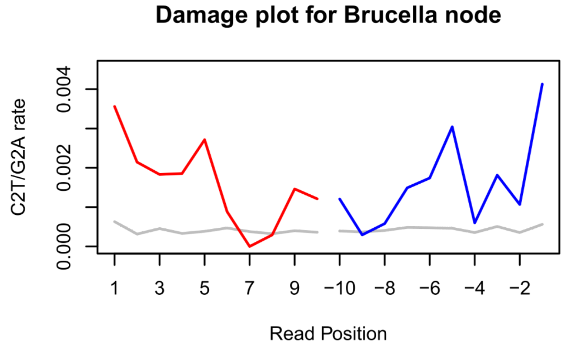

Node	Brucella
Top Reference	Brucella_melitensis
all reads	20156
nonDup	20040
readDis	0.557
nonStacked	6842
destacking?	On
C>T_1	0.0036
G>A_−1	0.0041
mean length (sd)	52 (17.568)

Brucella_melitensis;_NZ_CP022827.1 100%
Brucella_melitensis_bv._2_str._63/9;_NZ_CP007789.1 099%
Brucella_abortus_bv._6_str._870;_NZ_CP007709.1 098%
Brucella_abortus;_NZ_CP025743.1 098%
Brucella_abortus;_NZ_CP007738.1 098%
Brucella_suis;_NZ_CP007717.1 098%
Brucella_abortus;_NZ_CP007682.1 098%
Brucella_abortus;_NZ_CP044338.1 098%
Brucella_abortus;_NZ_CP022879.1 098%
Brucella_sp._2002734562;_NZ_CP016979.1 098%

Supplementary Figure 5: HOPS multi-panel identification. The green bar plot on the top left of the figure is representing the edit distance of the reads assigned to the *Brucella* node which are not showing damage. The green bar plot on the top right of the figure is representing the edit distance of the reads assigned to the *Brucella* node which are carrying damage. The damage across all reads are represented in the damage plot in the middle of the figure. The table in the bottom of the figure represents the top 10 Reference Genomes assigned to interrogated nodes. The percentage values in the table contain a superfluous zero i.e. 099% = 99%.

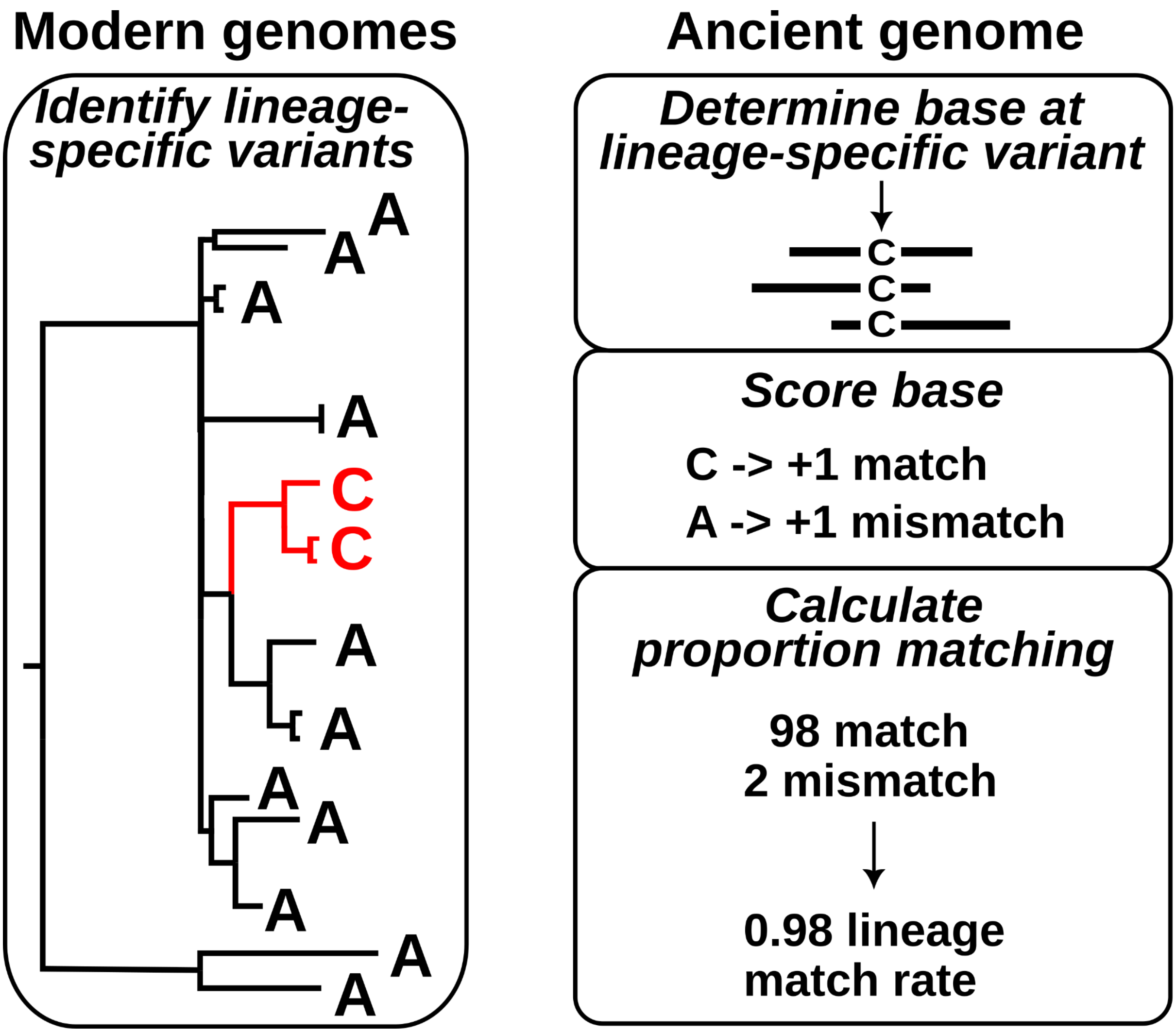

Supplementary Figure 6: Explanation of the lineage based species detection method. We create a phylogeny of genomes and then identify variant sites which define or are diagnostic of specific lineages. In the example of the left plot, all members of the red lineage carry “C” at a site in the genome, while all other genomes carry “A” i.e. “C” is the lineage-specific variant. We then assess lineage-specific/diagnostic sites in the ancient sample, calculating the frequency which the ancient genome matches the lineage-specific variant.

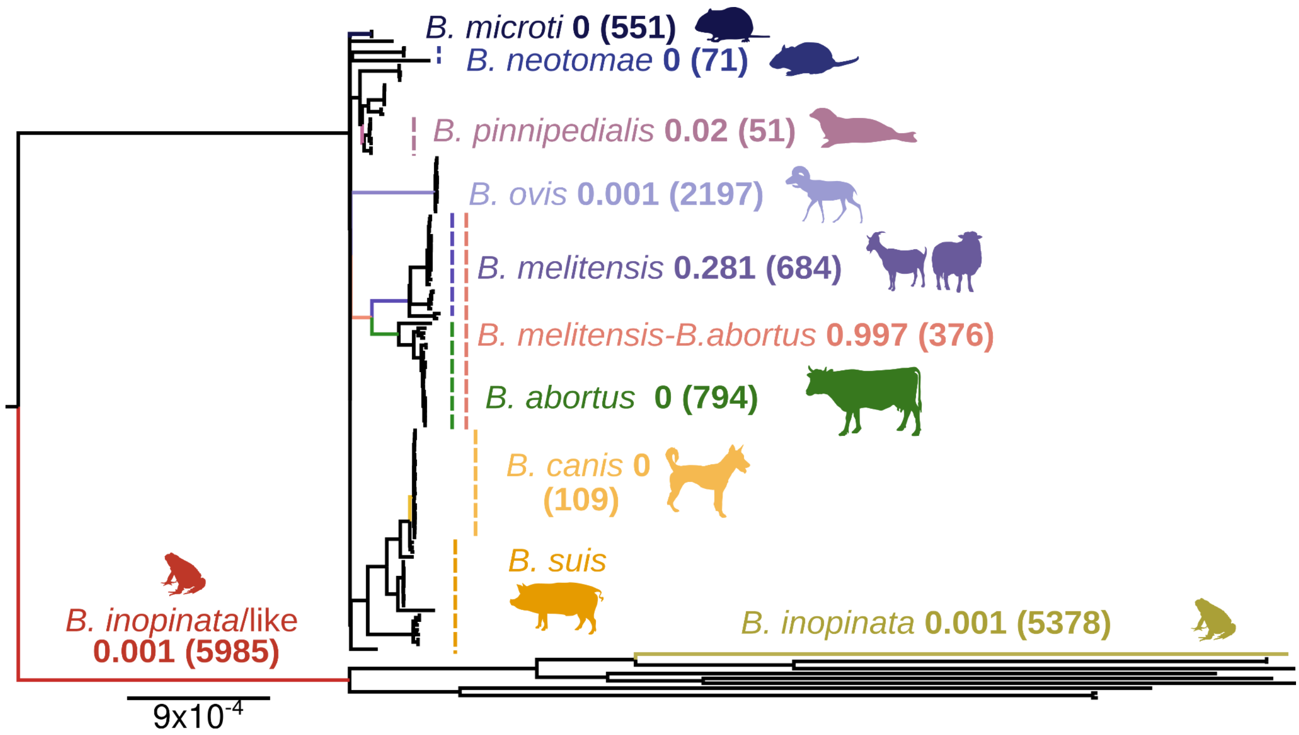

Supplementary Figure 7: Figurative representation of the lineage variant analysis for Mentese6, using data aligned to *B. melitensis*. Mentese6 carries ~100% of variants which define the *B. melitensis*-*B. abortus* clade, and ~28% of variants defining the *B. melitensis* clade. In comparison the alignment contains a small number of variants defining other clades.

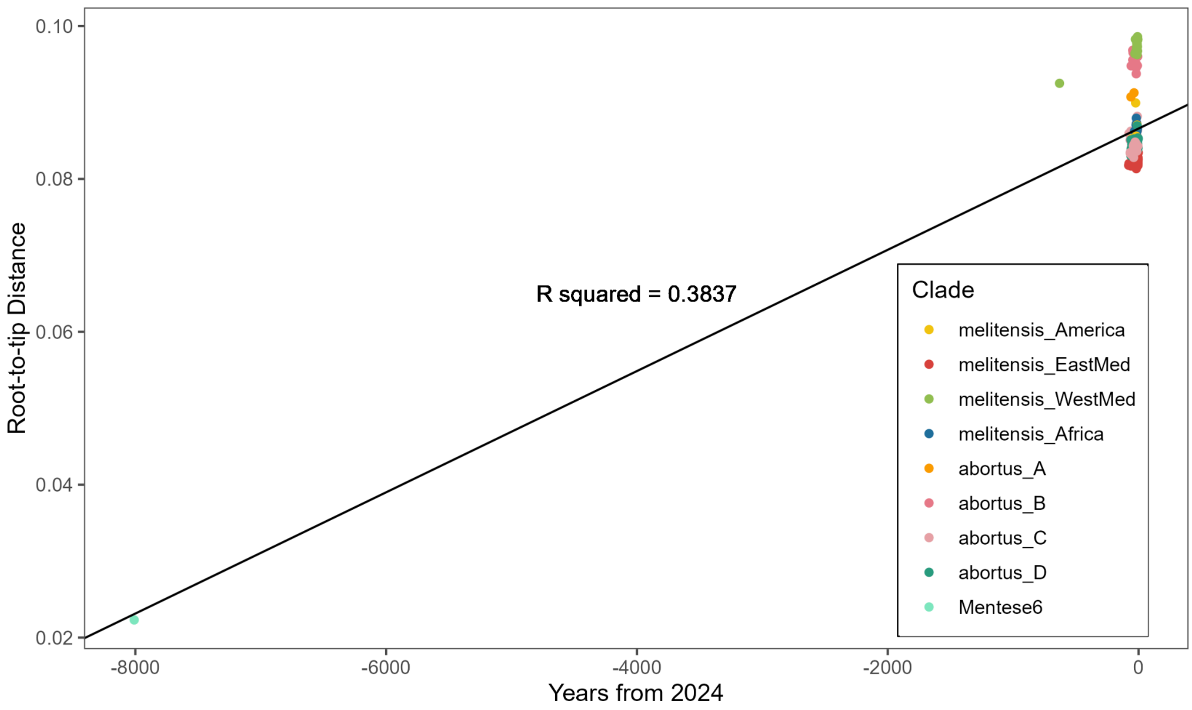

Supplementary Figure 8: Root-to-tip distances measuring temporal signal in *B. abortus* and *B. melitensis* clades, as computed by TempEst. R^2, slope and intercept were obtained from heuristic residual mean squared method. There is a presence of a weak temporal signal (R^2 = 0.3837) in the total dataset but it is confounded by local clock rates specific to subclades as expressed by clustering of clades by their root-to-tip distance.
