# Supplementary material for: An 8000 years old genome reveals the Neolithic origin of the zoonosis Brucella melitensis
Source: Nat Commun. 2024 Jul 20;15:6132. doi: 10.1038/s41467-024-50536-1 (PMC11271283; doi:10.1038/s41467-024-50536-1)
Supplement: Supplementary file 4 — Reporting Summary [file 41467_2024_50536_MOESM4_ESM.pdf]

nature portfolio

Corresponding author(s): Kevin G Daly

Last updated by author(s): 07/10/2024

Reporting Summary

Nature Portfolio wishes to improve the reproducibility of the work that we publish. This form provides structure for consistency and transparency in reporting. For further information on Nature Portfolio policies, see our Editorial Policies and the Editorial Policy Checklist.

Statistics

For all statistical analyses, confirm that the following items are present in the figure legend, table legend, main text, or Methods section.

n/a	Confirmed	
☐	☒	The exact sample size (*n*) for each experimental group/condition, given as a discrete number and unit of measurement
☒	☐	A statement on whether measurements were taken from distinct samples or whether the same sample was measured repeatedly
☐	☒	The statistical test(s) used AND whether they are one- or two-sided<br>*Only common tests should be described solely by name; describe more complex techniques in the Methods section.*
☒	☐	A description of all covariates tested
☒	☐	A description of any assumptions or corrections, such as tests of normality and adjustment for multiple comparisons
☐	☒	A full description of the statistical parameters including central tendency (e.g. means) or other basic estimates (e.g. regression coefficient) AND variation (e.g. standard deviation) or associated estimates of uncertainty (e.g. confidence intervals)
☐	☒	For null hypothesis testing, the test statistic (e.g. *F*, *t*, *r*) with confidence intervals, effect sizes, degrees of freedom and *P* value noted<br>*Give P values as exact values whenever suitable.*
☐	☒	For Bayesian analysis, information on the choice of priors and Markov chain Monte Carlo settings
☒	☐	For hierarchical and complex designs, identification of the appropriate level for tests and full reporting of outcomes
☒	☐	Estimates of effect sizes (e.g. Cohen's *d*, Pearson's *r*), indicating how they were calculated

Our web collection on statistics for biologists contains articles on many of the points above.

Software and code

Policy information about availability of computer code

Data collection	No specialized code was employed for data retrieval. All comparative data for this study were gathered from previously published works (as listed in the Methods section) and downloaded from public databases like the National Center for Biotechnology Information (NCBI) and the European Nucleotide Archive (ENA). Accessions used are provided in Supplementary Data 6.
Data analysis	The softwares used in this study are the following:<br>Oxcal v4.4 (Ramsey, 2001); Fastq Screen v0.15.2 (Wingett & Andrews, 2018); Prinseq v1.2 (Schmieder & Edwards, 2011); AdapterRemoval v2.3.2 (Schubert et al., 2016); bowtie v2.5.1 (Poullet & Orlando, 2020); KrakenUniq v1.0.4 (Breitwieser et al., 2018); HOPS v0.33 (Hübler et al., 2019); snippy (https://github.com/tseemann/snippy); treemmer_v0.3 (https://github.com/fmenardo/Treemmer) ; IQTREE-2 (Minh et al., 2020); bwa v0.7.18-r1243-dirty (Li & Durbin, 2010); samtools v1.19.2 (Danecek et al., 2021); MarkDuplicates v2.26.11 (https://broadinstitute.github.io/picard/); mapDamage v2.2.1 (Jónsson et al., 2013); bedtools v2.31.1 (Quinlan & Hall, 2010); ART v2.5.8 (Huang et al., 2012); GATK v3.5 (DePristo et al., 2011); MultivcfAnalyzer v0.85 (https://github.com/alexherbig/MultiVCFAnalyzer, Bos et al., 2014); MALT v040 (Vagene et al., 2018); bcftools v1.19 (https://samtools.github.io/bcftools/bcftools.html) ;vcftools v0.1.16 (https://vcftools.sourceforge.net/); seqkit v2.7.0 (Shen et al., 2016); ANIclustermap (https://github.com/moshi4/ANIclustermap); TempEst v1.5.3 (http://tree.bio.ed.ac.uk/software/tempest/); Gubbins (Croucher et al., 2015); BEAST v2.6.7 (Bouckaert et al., 2019); Tracer v1.7.2 (http://tree.bio.ed.ac.uk/software/tracer/); ; LogCombiner v2.6.7 (Bouckaert et al., 2019). We also used custom scripts available at https://github.com/LouisLhote/Neolithic_Brucella_paper/ and doi:10.5072/zenodo.80001

For manuscripts utilizing custom algorithms or software that are central to the research but not yet described in published literature, software must be made available to editors and reviewers. We strongly encourage code deposition in a community repository (e.g. GitHub). See the Nature Portfolio guidelines for submitting code & software for further information.

Data

Policy information about availability of data

All manuscripts must include a data availability statement. This statement should provide the following information, where applicable:

- Accession codes, unique identifiers, or web links for publicly available datasets
- A description of any restrictions on data availability
- For clinical datasets or third party data, please ensure that the statement adheres to our policy

Collapsed sequencing data with host reads removed and B. melitensis-aligned bam file for Mentese prior to data QC are available at ENA accession PRJEB75678 [https://www.ebi.ac.uk/ena/browser/view/PRJEB75678]. Reference sequences used in this study are previously published and available for B. melitensis [https://www.ncbi.nlm.nih.gov/datasets/genome/GCF_000007125.1/], B. abortus [https://www.ncbi.nlm.nih.gov/datasets/genome/GCF_000054005.1/], and B. suis [https://www.ncbi.nlm.nih.gov/datasets/genome/GCF_000007505.1/].

We employed sequences previously analyzed by Abdel-Glil and colleagues [https://doi.org/10.1128/jcm.00311]; ENA accessions for each are presented in Supplementary Data 6. Source data are provided with this paper.

Research involving human participants, their data, or biological material

Policy information about studies with human participants or human data. See also policy information about sex, gender (identity/presentation), and sexual orientation and race, ethnicity and racism.

Reporting on sex and gender	n.a.
Reporting on race, ethnicity, or other socially relevant groupings	n.a.
Population characteristics	n.a.
Recruitment	n.a.
Ethics oversight	n.a.

Note that full information on the approval of the study protocol must also be provided in the manuscript.

Field-specific reporting

Please select the one below that is the best fit for your research. If you are not sure, read the appropriate sections before making your selection.

☒ Life sciences ☐ Behavioural & social sciences ☐ Ecological, evolutionary & environmental sciences

For a reference copy of the document with all sections, see nature.com/documents/nr-reporting-summary-flat.pdf

Life sciences study design

All studies must disclose on these points even when the disclosure is negative.

Sample size	Sample size for newly screened specimens was determined by the number of available sheep/goat temporal bone remains from Menteşe Höyük. For screening of published ancient ruminant datasets, we screened one fastq file for each available specimen on ENA at the time of writing. We generated only a single Brucella melitensis genome as only one screened specimen passed our metagenomic threshold for detection. For HOPS screening of sequencing libraries, we screened the initial seven sequencing libraries available due to computation limits imposed by MALT. For the "lineage analysis" dataset, genomes for each species (Abdel-Glil, M. Y. et al., 2022) were randomly downsampled to a maximum of 23 representatives, to the same number of genomes as Brucella canis, to avoid lineage oversampling (see Supplementary Data S6).
Data exclusions	For the "phylogenetic analysis and reference datasets" (Supplementary Data 5) we excluded Brucella melitensis and Brucella abortus data (Abdel-Glil, M. Y. et al., 2022) which lacked a sampling year, to ensure minimal uncertainty on the temporal age of samples. We additionally thinned the Brucella melitensis-abortus data set using treemmer_v0.3, following (Long et al, 2023), using a relative tree length of 95% (-RTL 0.95). We excluded genome sites in Mentes6 which did not pass our criteria (3-7 or 3-6 reads for chromosome I and II, 100% base concordance) and for the Beast analysis we excluded regions detected to experience recombination by Gubbins and also with any missing data in modern sequences; see Methods for full data.
Replication	No biological replicates were available due to the unique nature of the archaeological material. Multiple PCR amplifications were performed on several dsDNA sequencing libraries prepared from the densest Mentese6 specimen powder, which all were positive for the presence of Brucella melitensis-aligning reads with post-mortem damage. For C14 dating, two replicates were performed at different radiocarbon dating labratories (Supplementary Data S3) For PCR-indexing amplifications, we performed a total of 51 individual amplifications, which retained post-mortem damage in terminal base pairs (Supplementary Data 4). Three replicates for the Beast model best fitting the data was run, and

	the chain merged using logcombiner. The phylogenetic position of the Mentese6 sequence was assessed using multiple complementary approaches (ML phylogeny, diagnostic variants, Beast phylogeny).
Randomization	No randomization testing was possible as the study utilizes shotgun sequencing to analyze archaeological remains in an exploratory fashion.
Blinding	Blinding was not applied in this study as it was exploratory in nature; it was unknown which of or if any of the Mentese specimens or published ruminant datasets were positive for ancient Brucella DNA.

Reporting for specific materials, systems and methods

We require information from authors about some types of materials, experimental systems and methods used in many studies. Here, indicate whether each material, system or method listed is relevant to your study. If you are not sure if a list item applies to your research, read the appropriate section before selecting a response.

Materials & experimental systems

n/a	Involved in the study	
☒	☐	Antibodies
☒	☐	Eukaryotic cell lines
☐	☒	Palaeontology and archaeology
☒	☐	Animals and other organisms
☒	☐	Clinical data
☒	☐	Dual use research of concern
☒	☐	Plants

Methods

n/a	Involved in the study	
☒	☐	ChIP-seq
☒	☐	Flow cytometry
☒	☐	MRI-based neuroimaging

Palaeontology and Archaeology

Specimen provenance	The faunal material from Menteşe Höyük, Northwest Türkiye, from the 2000 test pits was exported in 2002 to Lionel Gourichon with the permission of the director of the Iznik Museum, Taylan Sevil, and the site's excavation directors, Jacob Roodenberg and Songül Alpaslan-Roodenberg. Consent for the genetic analysis, as part of the ERC CODEX project, was given in August 2013 by Ömer Eren , then director of the Iznik Museum, with the approval of the excavation directors
Specimen deposition	The screened Menteşe specimens are under the temporary stewardship of Kevin G. Daly (UCD, Dublin) and permanent stewardship of Lionel Gourichon, Université Côte d'Azur, Nice. Applications to access and re-examine the material should be made to Dr. Gourichon. Specimen identifiers are provided in Data S1.
Dating methods	Dating of the Mentese6 sample was performed by two C14 laboratories: ChronoCentre, QUB, Belfast (UBA-47124) and ORAU, Oxford (OxA-43559). Sample pretreatment and measure was according to standard practices of these laboratories. Dating was performed on the sample bone powder (subsampled) as was subjected to DNA extraction. Uncalibrated dates (Supplementary Data 3) were calibrated with Oxcal v4.4 using IntCal20, reporting a confirmed date after testing for internal consistency.

☒ Tick this box to confirm that the raw and calibrated dates are available in the paper or in Supplementary Information.

Ethics oversight	This study used material excavated for the purposes of archaeozoological analysis and did not require additional excavation work. The Mentese specimens were analyzed with permission from the custodian Lionel Gourichon who is also a co-author of this study.

Note that full information on the approval of the study protocol must also be provided in the manuscript.

Plants

Seed stocks	n.a.
Novel plant genotypes	n.a.
Authentication	n.a.
